# Validity of Self-Reports of Height and Weight among the General Adult Population in Japan: Findings from National Household Surveys, 1986

**DOI:** 10.1371/journal.pone.0148297

**Published:** 2016-02-10

**Authors:** Nayu Ikeda

**Affiliations:** Center for International Collaboration and Partnership, National Institute of Health and Nutrition, National Institutes of Biomedical Innovation, Health and Nutrition, Shinjuku, Tokyo, Japan; Leibniz Institute for Prevention Research and Epidemiology (BIPS), GERMANY

## Abstract

**Background/Objectives:**

Growing evidence indicates that self-reported height and weight are biased, but little is known about systematic errors in the general adult population in Japan. This study takes advantage of the unique opportunity to examine this issue provided by the 1986 National Nutrition Survey.

**Subjects/Methods:**

Individual-level data on a nationally representative sample aged 20–89 years from the National Nutrition Survey (November 1986) were merged with Comprehensive Survey of Living Conditions (September 1986) data to obtain a dataset containing both self-reported and measured data on height and weight for each person (*n* = 10,469). Discrepancies between self-reported and measured means of height, weight, and body mass index (BMI) were tested across measured BMI categories (<18.5, 18.5–24.9, 25.0–27.4, 27.5–29.9, and ≥30.0 kg/m^2^), age groups (20–44, 45–64, and 65–89 years), and sexes. Reporting bias in mean BMI was decomposed into the contributions of misreporting height and weight. The sensitivity and specificity of self-reported BMI categories were estimated.

**Results:**

Mean self-reported BMI was substantially underestimated in older women (P<0.001; Cohen’s d, -0.4), and the major contributor to the bias was their over-reported height. Mean self-reported BMI was also considerably underestimated in both men and women who were overweight and obese (P<0.001; Cohen’s d, -1.0 to -0.6), due mainly to their underreported weight. In contrast, mean self-reported BMI was considerably overestimated in underweight men (P<0.001; Cohen’s d, 0.5), due largely to their over-reported weight. The sensitivity of self-reported BMI categories was particularly low for individuals who had a measured BMI of 27.5–29.9 kg/m^2^ (40.9% for men and 26.8% for women).

**Conclusions:**

Self-reported anthropometric data were not sufficiently accurate to assert the validity of their use in epidemiological studies on the general adult population in Japan in the late 1980s.

## Introduction

The demand for collecting accurate anthropometric data at the population level has been growing worldwide. Many countries conduct a physical examination of height and weight as part of their national health surveys[1] to obtain an objective nutritional status profile of a population. Although a direct measurement provides the most accurate estimates of height and weight, it requires considerable standardization and quality control efforts to be implemented at the national level on a regular basis. Given this limitation of physical measurements, self-reports serve as a relatively convenient alternative for large population surveys, although it is well established that body height and weight tend to be over-reported and underreported, respectively.[2] Such systematic errors inherent in self-reported data can bias estimates of health and mortality risks related to body mass index,[3, 4] leading to erroneous inferences on the impacts of public health policies and interventions.

Previous studies have analyzed national health surveys from several industrialized countries to explore reporting biases in height and weight at the population level.[5–10] In Japan, a few studies have examined the validity of self-reported height and weight, but their subjects have been limited to narrow groups such as female employees of a private company,[11] middle-aged local government officials,[12] and a nationally representative sample of older people.[13] These studies concluded that in Japan, self-reported height, weight, and body mass index (BMI) are generally reliable and accurate, and that these measurements are consequently usable for epidemiological surveys. However, no study has examined the reporting bias in anthropometric data for the general adult population covering the entire age range and its variation across subgroups in this country.

The National Nutrition Survey (NNS) and its successor, the National Health and Nutrition Survey, are the major source of anthropometric data on the population of Japan since the late 1940s. In these surveys, height and weight of participants are principally measured at a physical examination site by trained personnel and in accordance with a standardized protocol.[14] However, participants may self-report their height and weight, which are supposedly measured at home, if it is not convenient for them to have a direct measurement taken at a physical examination site. Until the 2012 survey, the questionnaire did not record whether the measurement of height and weight of a participant was a self-report.[14] Consequently, there is concern about whether self-reported height and weight are as accurate as direct measurements or whether this type of measurement has biased population estimates of overweight and obesity in Japan.

Although it is not possible to assess this question directly, the 1986 NNS provides the unique opportunity to merge measured data with the corresponding self-reports of participants obtained from its master sample used in the Comprehensive Survey of Living Conditions (CSLC). The NNS measured height and weight annually, while it was only in 1986 that the CSLC asked participants to self-report their height and weight. No previous study has analyzed these surveys to examine this topic for almost 30 years, perhaps because little was known among researchers about the availability of data on self-reported height and weight from the 1986 CSLC. Taking advantage of this unique opportunity provided by the 1986 surveys, the author examined the reliability of self-reported height and weight in the general adult population in Japan.

## Materials and Methods

### Ethics Statement

This research was a cross-sectional study involving secondary analysis of observational survey data. Under the Statistics Act,[15] the Ministry of Health, Labour and Welfare anonymized individual-level data collected from the NNS and the CSLC and provided the author with the datasets for this study. In accordance with the Ethical Guidelines of Epidemiological Research established by the Ministry of Education, Culture, Sports, Science and Technology and the Ministry of Health, Labour and Welfare,[16] these guidelines were not applicable to this study because it used only information that had already been anonymized at the time of the study.

### Data sources

This analysis was based on data from the NNS and the CSLC, conducted by the Ministry of Health in 1986. The NNS began in the late 1940s as an annual cross-sectional household interview and examination survey, and it currently continues as the National Health and Nutrition Survey, collecting data on diet, physical activity, lifestyles, anthropometric measures, and biochemical and clinical profiles.[14] The CSLC started in 1986 as an annual cross-sectional household interview survey to collect information on the socioeconomic status and living conditions of the population, and it includes an additional survey component on health status every 3 years.[17]

The 1986 NNS used a stratified two-stage cluster sampling design to obtain a nationally representative sample of the non-institutionalized Japanese population. The sampling frame was the list of all residential enumeration areas that was defined for the 1985 Population Census and stratified into 47 prefectures. Each census enumeration area consisted of approximately 50 households. In the first sampling stage, a simple random sample of census enumeration areas was drawn from each prefecture. All members of approximately 240,000 households in 4,966 sampled census enumeration areas were eligible for the CSLC, which was conducted on September 4, 1986.[18] The second sampling stage for the NNS was implemented in November 1986; the census enumeration areas chosen for the CSLC were divided into unit blocks such that each block consisted of 20–30 households. Using simple random sampling, unit blocks were then sampled from each prefecture to total 300. After excluding households not participating in the CSLC and those moving into the sampled unit blocks after the CSLC, individuals aged 1 year or older living in approximately 7,000 households were eligible for the NNS. A sampled household was excluded from the survey if the head of the household was non-Japanese. Groups of individuals who did not share either living quarters or living expenses were considered a single household if they regularly shared meals.[19]

### Measurement

Participants aged 20 years and over in the 1986 CSLC filled in a self-administered questionnaire at home, self-reporting their height and weight in centimeters and kilograms, respectively, to the nearest integer.[18] They did not know at the time of the CSLC that they would have their height and weight directly measured if they were sampled for the NNS. In the NNS, participants aged 1 year and older were asked to attend a physical examination held at a local community center near their residence. Height was measured barefoot with a stadiometer to the nearest millimeter. Weight was measured in light clothing with a scale to the nearest 0.1 kg. If participants preferred their weight to be measured while full dressed, they were asked to weigh their clothes at home in advance so that this weight could be subtracted from the body weight measured in the physical examination.[19]

BMI was calculated in this study as weight in kilograms divided by the square of height in meters. Measured BMI was based on measured values of height and weight from the NNS. Self-reported BMI was based on self-reported values of height and weight from the CSLC. Participants were classified by self-reported and measured BMI into “underweight” (< 18.5 kg/m^2^), “normal weight” (18.5–24.9 kg/m^2^), “overweight” (25.0–27.4 and 27.5–29.9 kg/m^2^), and “obese” (≥ 30.0 kg/m^2^).

### Study population

Individual records of the NNS were merged with those of the CSLC through deterministic record linkage to obtain a dataset that contained both self-reported and direct measures of height and weight for each person in the sample. The analytic sample of this study was limited to participants aged 20–89 years at the time of the NNS. Of 17,071 NNS participants, 5,303 aged < 20 or ≥ 90 years, 117 pregnant participants, and 6 twins were excluded from the analysis. Of the remaining records of 11,645 participants, 10,640 were merged between the NNS and the CSLC by key variables on prefecture of residence, masked identification numbers of survey blocks and households, sex, year and month of birth. The final analytic sample consisted of 10,469 participants (4,599 men and 5,870 women), after excluding 170 participants with missing data on measured or self-reported height or weight (only self-reported data missing for 158, only measured data missing for 8, and both missing for 4) and 1 participant with an outlier on self-reported weight.

### Statistical analysis

Mean population height, weight and BMI were estimated using both measured and self-reported data by weight status, age group, and sex. A difference in means was calculated by subtracting an average of measured data from that of self-reported data. Paired-samples *t* tests were used to examine whether differences between measured and self-reported means were equal to zero. However, tests on the large sample might declare negligible differences to be significant.[20, 21] Therefore, Cohen’s *d* statistics was calculated to assess the meaningfulness of the differences: it is a standardized measure of the difference between the two means divided by the standard deviation. Cohen’s *d*s of 0.2, 0.5, and 0.8 indicate small, medium, and large differences, respectively.[22] Moreover, a difference between self-reported BMI and measured BMI for each participant was decomposed into differences in height and weight. The difference in BMI was mathematically expanded into three components: (1) the self-report bias in height multiplied by self-reported weight; (2) the self-report bias in weight multiplied by the inverse of the square self-reported height; and (3) the product of the self-report bias in height and that in weight, as shown in the following equation:

$$BMI_{R,i}-BMI_{M,i}=W_{R,i}\left(H_{R,i}{}^{-2}-H_{M,i}{}^{-2}\right)+H_{R,i}{}^{-2}\left(W_{R,i}-W_{M,i}\right)-\left(H_{R,i}{}^{-2}-H_{M,i}{}^{-2}\right)\left(W_{R,i}-W_{M,i}\right)$$

where the subscript *i* denotes individual participants, *H* and *W* stand for height and weight, respectively, and subscripts *R* and *M* indicate self-reported and measured values, respectively. The third interaction term in the equation was considered negligibly small. Using this equation, contributions of self-report bias in height and weight to the self-report bias in BMI were computed for each individual and then aggregated across individuals to obtain national estimates.

Sensitivity and specificity of the classification of BMI based on self-reports were assessed against the classification of measured BMI used as the gold standard. Sensitivity was defined as the probability that participants classified in a given BMI category based on measured BMI would be classified in the same category based on self-reported values (true positives). Specificity was defined as the probability that participants who were not classified in a given BMI category on the basis of measured values would not be classified in that category based on self-reported values (true negatives). All analyses were conducted using Stata version 11.0 (StataCorp, College Station, Texas, USA). A P-value of less than 0.05 was considered statistically significant.

## Results

On average, both men and women significantly over-reported their height by 0.9 cm and 1.4 cm, respectively (P < 0.001), while Cohen’s *d*s indicate that these differences were small (Table 1). Mean self-reported height was significantly higher than mean measured height in both sexes across groups defined by age and measured BMI (P < 0.001), except for obese men (P = 0.073). The difference in means was medium for both men and women who were aged 45–89 years or overweight and women who were obese.

**Table 1:** Differences between self-reported and measured means of height by characteristics of participants based on the National Nutrition Survey, 1986.

Characteristics	N	Self-reported		Measured		Difference ^a^		P-value	Cohen’s d
Men									
Overall	4,599	165.6	(0.1)	164.7	(0.2)	0.9	(0.0)	<0.001	0.3
Age, years									
20–44	2,200	168.7	(0.1)	168.1	(0.1)	0.6	(0.1)	<0.001	0.2
45–64	1,716	163.9	(0.2)	162.9	(0.2)	1.0	(0.1)	<0.001	0.4
65–89	683	160.2	(0.3)	158.3	(0.3)	1.9	(0.1)	<0.001	0.5
Measured BMI, kg/m^2^									
< 18.5	288	164.6	(0.5)	163.7	(0.5)	0.9	(0.2)	<0.001	0.3
18.5–24.9	3,409	165.7	(0.2)	164.8	(0.2)	0.9	(0.1)	<0.001	0.3
25.0–27.4	678	165.7	(0.3)	164.5	(0.3)	1.2	(0.1)	<0.001	0.4
27.5–29.9	176	166.2	(0.5)	164.9	(0.5)	1.2	(0.2)	<0.001	0.5
≥ 30.0	48	165.8	(0.9)	164.6	(0.8)	1.2	(0.7)	0.073	0.3
Women									
Overall	5,870	153.3	(0.1)	151.9	(0.1)	1.4	(0.1)	<0.001	0.3
Age, years									
20–44	2,789	155.7	(0.1)	155.1	(0.1)	0.7	(0.0)	<0.001	0.3
45–64	2,195	152.1	(0.1)	150.6	(0.2)	1.5	(0.1)	<0.001	0.4
65–89	886	148.5	(0.2)	145.3	(0.3)	3.2	(0.2)	<0.001	0.7
Measured BMI, kg/m^2^									
< 18.5	521	154.3	(0.3)	153.4	(0.4)	0.9	(0.2)	<0.001	0.3
18.5–24.9	4,131	153.5	(0.1)	152.3	(0.2)	1.2	(0.1)	<0.001	0.3
25.0–27.4	763	152.1	(0.2)	150.3	(0.2)	1.8	(0.1)	<0.001	0.5
27.5–29.9	306	152.1	(0.3)	149.7	(0.4)	2.5	(0.2)	<0.001	0.7
≥ 30.0	149	152.8	(0.5)	150.3	(0.5)	2.5	(0.4)	<0.001	0.5

Abbreviation: BMI, body mass index. Values in parentheses denote standard errors.

^a^ Calculated by subtracting measured values from self-reported values.

Mean self-reported weight was significantly lower than mean measured weight by 0.2 kg in men (P = 0.001) and 0.6 kg in women (P < 0.001, Table 2), while according to Cohen’s *d*s, these differences were small. Mean self-reported weight was significantly different from mean measured weight across age groups except women aged 65–89 years, while the differences were small. Mean weight was also significantly underreported in both men and women who were overweight or obese. In contrast, the mean self-reported weight was significantly higher than the mean measured weight among people who had a measured BMI < 18.5 kg/m^2^ (2.4 kg for men and 1.1 kg for women, P < 0.001). The difference in means was large in obese women, and it was medium in underweight, overweight and obese men and overweight women.

**Table 2:** Differences between self-reported and measured means of weight by characteristics of participants based on the National Nutrition Survey, 1986.

Characteristics	N	Self-reported		Measured		Difference ^a^		P-value	Cohen’s d
Men									
Overall	4,599	61.3	(0.2)	61.5	(0.2)	-0.2	(0.1)	0.001	-0.1
Age, years									
20–44	2,200	63.5	(0.2)	63.8	(0.2)	-0.3	(0.1)	<0.001	-0.1
45–64	1,716	61.1	(0.2)	61.3	(0.2)	-0.2	(0.1)	0.011	-0.1
65–89	683	55.0	(0.4)	54.7	(0.4)	0.3	(0.2)	0.044	0.1
Measured BMI, kg/m^2^									
< 18.5	288	49.5	(0.4)	47.1	(0.4)	2.4	(0.2)	<0.001	0.6
18.5–24.9	3,409	59.7	(0.2)	59.7	(0.2)	0.0	(0.1)	0.729	0.0
25.0–27.4	678	69.1	(0.3)	70.6	(0.2)	-1.5	(0.1)	<0.001	-0.4
27.5–29.9	176	75.6	(0.6)	77.4	(0.5)	-1.8	(0.2)	<0.001	-0.6
≥ 30.0	48	81.9	(1.1)	85.1	(0.9)	-3.2	(0.9)	0.001	-0.5
Women									
Overall	5,870	51.4	(0.1)	52.0	(0.1)	-0.6	(0.0)	<0.001	-0.2
Age, years									
20–44	2,789	51.6	(0.1)	52.4	(0.1)	-0.8	(0.1)	<0.001	-0.3
45–64	2,195	52.5	(0.2)	53.0	(0.2)	-0.5	(0.1)	<0.001	-0.2
65–89	886	48.1	(0.3)	48.2	(0.3)	0.0	(0.2)	0.791	0.0
Measured BMI, kg/m^2^									
< 18.5	521	42.4	(0.2)	41.3	(0.2)	1.1	(0.2)	<0.001	0.3
18.5–24.9	4,131	50.1	(0.1)	50.4	(0.1)	-0.3	(0.0)	<0.001	-0.1
25.0–27.4	763	57.7	(0.2)	59.0	(0.2)	-1.3	(0.1)	<0.001	-0.4
27.5–29.9	306	61.2	(0.3)	64.0	(0.3)	-2.8	(0.2)	<0.001	-0.6
≥ 30.0	149	67.2	(0.7)	73.1	(0.6)	-5.9	(0.6)	<0.001	-0.8

Abbreviation: BMI, body mass index. Values in parentheses denote standard errors.

^a^ Calculated by subtracting measured values from self-reported values.

The mean self-reported BMI was significantly lower than the mean measured BMI by 0.3 kg/m^2^ in men and 0.7 kg/m^2^ in women (P < 0.001), while these differences were small (Table 3). Mean BMI was significantly underreported compared with measured BMI in all sex-age groups. By the classification of measured BMI, mean BMI was significantly underreported among individuals with a BMI of ≥ 18.5 kg/m^2^ (P < 0.001), while it was over-reported among those who were underweight (P < 0.001). The difference in means was large in both men and women who had a measured BMI ≥ 27.5 kg/m^2^; and it was medium in women aged 45–89 years, underweight men, and both men and women who had a measured BMI of 25.0–27.4 kg/m^2^. These medium and large differences were largely accounted for by over-reported height in women aged 45–89 years, over-reported weight in underweight men, and underreported weight in both men and women who were overweight or obese (Table 4).

**Table 3:** Differences between self-reported and measured means of body mass index based on the National Nutrition Survey, 1986^a^.

Characteristics	N	Self-reported		Measured		Difference ^b^		P-value	Cohen’s d
Men									
Overall	4,599	22.3	(0.0)	22.6	(0.0)	-0.3	(0.0)	<0.001	-0.2
Age, years									
20–44	2,200	22.3	(0.1)	22.6	(0.1)	-0.3	(0.0)	<0.001	-0.2
45–64	1,716	22.7	(0.1)	23.0	(0.1)	-0.3	(0.0)	<0.001	-0.3
65–89	683	21.4	(0.1)	21.8	(0.1)	-0.4	(0.1)	<0.001	-0.2
Measured BMI, kg/m^2^									
< 18.5	288	18.2	(0.1)	17.5	(0.1)	0.7	(0.1)	<0.001	0.5
18.5–24.9	3,409	21.7	(0.0)	22.0	(0.0)	-0.2	(0.0)	<0.001	-0.2
25.0–27.4	678	25.2	(0.1)	26.1	(0.0)	-0.9	(0.1)	<0.001	-0.6
27.5–29.9	176	27.3	(0.1)	28.4	(0.0)	-1.1	(0.1)	<0.001	-0.8
≥ 30.0	48	29.7	(0.3)	31.4	(0.2)	-1.6	(0.3)	<0.001	-0.9
Women									
Overall	5,870	21.9	(0.0)	22.5	(0.1)	-0.7	(0.0)	<0.001	-0.3
Age, years									
20–44	2,789	21.3	(0.1)	21.8	(0.1)	-0.5	(0.0)	<0.001	-0.3
45–64	2,195	22.7	(0.1)	23.4	(0.1)	-0.7	(0.0)	<0.001	-0.4
65–89	886	21.8	(0.1)	22.8	(0.1)	-1.0	(0.1)	<0.001	-0.4
Measured BMI, kg/m^2^									
< 18.5	521	17.8	(0.1)	17.5	(0.0)	0.3	(0.1)	<0.001	0.2
18.5–24.9	4,131	21.2	(0.0)	21.7	(0.0)	-0.5	(0.0)	<0.001	-0.3
25.0–27.4	763	24.9	(0.1)	26.1	(0.0)	-1.2	(0.1)	<0.001	-0.6
27.5–29.9	306	26.4	(0.1)	28.5	(0.0)	-2.1	(0.1)	<0.001	-1.0
≥ 30.0	149	28.8	(0.3)	32.3	(0.2)	-3.5	(0.3)	<0.001	-0.9

Abbreviation: BMI, body mass index. Values in parentheses denote standard errors.

^a^ Measured BMI was calculated using physical measurements of height and weight, and self-reported BMI was calculated using self-reported height and weight.

^b^ Calculated by subtracting measured values from self-reported values.

**Table 4:** Contributions of differences in mean height and weight to differences in mean body mass index (kg/m^2^) based on the National Nutrition Survey, 1986.

Characteristics	Height			Weight		
			P-value			P-value
Men						
Overall	-0.3	(0.0)	<0.001	-0.1	(0.0)	0.001
Age, years						
20–44	-0.2	(0.0)	<0.001	-0.1	(0.0)	<0.001
45–64	-0.3	(0.0)	<0.001	-0.1	(0.0)	0.008
65–89	-0.5	(0.0)	<0.001	0.1	(0.1)	0.040
Measured BMI, kg/m^2^						
< 18.5	-0.2	(0.0)	<0.001	0.9	(0.1)	<0.001
18.5–24.9	-0.2	(0.0)	<0.001	0.0	(0.0)	0.739
25.0–27.4	-0.4	(0.0)	<0.001	-0.5	(0.1)	<0.001
27.5–29.9	-0.4	(0.1)	<0.001	-0.7	(0.1)	<0.001
≥ 30.0	-0.4	(0.3)	0.083	-1.2	(0.4)	0.001
Women						
Overall	-0.4	(0.0)	<0.001	-0.2	(0.0)	<0.001
Age, years						
20–44	-0.2	(0.0)	<0.001	-0.3	(0.0)	<0.001
45–64	-0.5	(0.0)	<0.001	-0.2	(0.0)	<0.001
65–89	-1.0	(0.1)	<0.001	0.0	(0.1)	0.714
Measured BMI, kg/m^2^						
< 18.5	-0.2	(0.0)	<0.001	0.5	(0.1)	<0.001
18.5–24.9	-0.4	(0.0)	<0.001	-0.1	(0.0)	<0.001
25.0–27.4	-0.6	(0.0)	<0.001	-0.6	(0.1)	<0.001
27.5–29.9	-0.9	(0.1)	<0.001	-1.2	(0.1)	<0.001
≥ 30.0	-0.9	(0.2)	<0.001	-2.5	(0.3)	<0.001

Abbreviation: BMI, body mass index. Values in parentheses denote standard errors.

Sensitivity of self-reported BMI categories compared to measured BMI categories was 67.4% in men and 79.3% in women for those who were underweight (Table 5). It decreased to 40.9% in men and 26.8% in women for those who had a measured BMI of 27.5–29.9 kg/m^2^. Sensitivity was lower in women than in men for those who were overweight or obese. Specificity was above 95% for both men and women across the measured BMI categories except for those at a normal weight.

**Table 5:** Classification of survey participants according to measured and self-reported body mass index and test values of diagnosis based on self-reported values from the National Nutrition Survey, 1986.

Sex	Measured BMI (kg/m^2^)					
	< 18.5	18.5–24.9	25.0–27.4	27.5–29.9	≥ 30.0	Total
Men						
Self-reported BMI (kg/m^2^)						
< 18.5	194	95	0	0	0	289
18.5–24.9	93	3,209	275	11	2	3,590
25.0–27.4	1	98	379	87	3	568
27.5–29.9	0	6	23	72	18	119
≥ 30.0	0	1	1	6	25	33
Total	288	3,409	678	176	48	4,599
Sensitivity (%)	67.4	94.1	55.9	40.9	52.1	
Specificity (%)	97.8	68.0	95.2	98.9	99.8	
Women						
Self-reported BMI (kg/m^2^)						
< 18.5	413	224	1	0	1	639
18.5–24.9	105	3,827	375	65	18	4,390
25.0–27.4	2	71	349	148	25	595
27.5–29.9	1	6	33	82	54	176
≥ 30.0	0	3	5	11	51	70
Total	521	4,131	763	306	149	5,870
Sensitivity (%)	79.3	92.6	45.7	26.8	34.2	
Specificity (%)	95.8	67.6	95.2	98.3	99.7	

Abbreviation: BMI, body mass index.

## Discussion

To the best of the author’s knowledge, this study is the first to examine the validity of self-reported height and weight in the general adult population covering the entire age range in Japan. Results of this study confirm that in the late 1980s, mean BMI based on self-reported height and weight was substantially underestimated in older women due mainly to their over-reported height. It was also considerably underestimated in both men and women who were overweight and obese, and the major contributor to the bias was their underreported weight. In contrast, mean BMI based on self-reported height and weight was considerably overestimated in underweight men due largely to their over-reported weight.

The patterns of misreporting in height and weight across categories of actual BMI were consistent with those found in data from the National Health and Nutrition Examination Surveys in the United States in the 2000s.[23] A similar trend was also reported in a local cohort study on the middle-aged workplace population in Japan.[12] The substantial over-reporting of height among people aged 65–89 years in the present study may partly reflect their stooped posture. Moreover, findings on the discrepancy in directions of misreporting of body weight between the underweight group and heavier groups may partly reflect idiosyncratic factors, such as participants’ perceptions of ideal body weight.[24] In other words, people are likely to estimate their weight excessively toward standard body weight, regardless of whether they are underweight, overweight, or obese.

Using the nationally representative sample of the general adult population, this study demonstrated more detailed patterns of the significance and magnitude of misreporting of height and weight in comparison with results from previous studies on specific population groups in Japan. A study on female employees of a computer assembling factory in Fukushima Prefecture aged 20–42 years in 1995 showed that, with a time lag of 1 week between interview and examination, height and weight were significantly underreported by 0.1 cm and 0.2 kg, respectively, while misreporting of BMI was not significant.[11] The present study confirmed that women aged 20–44 years significantly over-reported their height and underreported their weight, while the magnitudes of their misreporting in height and weight were small. As a result, their mean BMI based on self-reports was significantly underestimated but the size of the bias was negligible. Moreover, a study on a large sample of public servants of a local government aged 35–64 years in 2002 showed that over-reporting of height was only significant among men at 0.08 cm, and no significant misreporting of weight was observed for either men or women.[12] In contrast, the present study showed that the over-reporting of height and underreporting of weight in the 45–64-year-old age group were significant for both men and women, while the underreporting of weight were small in size. The relatively large misreporting of height found in the present study might be partly explained by the use of a nationally representative sample of the general population covering all types of labor force status and job categories. Furthermore, the previous study on the elderly aged 70 years and over living in the community in 2009 showed significant over-reporting of height, by 0.9 cm for men and 1.2 cm for women, and significant underreporting of weight by 1.1 kg for men and 0.9 kg for women. In contrast, the present study demonstrated larger over-reporting of height but only marginal or insignificant underreporting of weight among people aged 65–89 years. These differences in results among older people between the present and previous studies are not attributable to the slight difference in age ranges between the two studies. It is not known whether reporting bias in height has decreased over time since the late 1980s in Japan, and further investigation is necessary to confirm the trends in reporting bias among the elderly based on a nationally representative sample.

Reflecting substantial misreporting of height and weight, this study suggests that the discriminant power of self-reported BMI for the detection of individuals with excess weight is not sufficient for claiming the validity of this measure’s use in epidemiological studies on the general adult population in Japan. In particular, considerable underreporting of weight among overweight and obese individuals casts doubt on the appropriateness of the current practice of the National Health and Nutrition Survey from a statistical point of view. Population estimates on nutritional status obtained from the survey might be biased through the self-reported height and weight of some participants. As it is not confirmed in the survey whether participants had their reported height and weight actually measured at home, the possibility of systematic errors in self-reports should always be kept in mind in the interpretation of results from this survey.

It is not known whether the accuracy of self-reported height and weight has improved in Japan since the late 1980s. Previous studies show that trends in the reporting bias in anthropometric data vary across countries. For example, underestimation of BMI by self-reports decreased in Australia from 1995 to 2008,[8] while it increased in Ireland from 1998 to 2007.[25] In the United States, underestimation of BMI diminished among obese people partly through changes in attitudes about obesity within society from 1988–1994 to 2005–2008, whereas it has been stable for underweight and normal-weight groups.[10] Another study also pointed out that underestimation of the prevalence of obesity had been constant in the United States from 1976 to 2004, while it had increased in Canada from 1986 to 2005.[6] To establish the validity of the use of self-reports in large population surveys, including the National Health and Nutrition Survey, it is necessary in future research to investigate how reporting bias for height and weight has changed since the late 1980s for the general adult population in Japan.

This study has several limitations. First, the surveys used in this study date back to the late 1980s, and the implications of the results might not be completely applicable to the present. However, as mentioned earlier, these surveys offer what is currently a unique opportunity for exploring the reporting bias of the general adult population at the national level in Japan, and the findings of this study add valuable knowledge to the literature. Second, although it was assumed in this study that there was no change in actual weight during the two months between the interview and the examination, this time gap might be sufficient for some participants to have had a substantial weight gain or loss, leading to overestimation of their misreporting of weight. Third, the 1986 NNS might have some participants self-report their height and weight instead of physical measurements, but this was not considered in this analysis because of the absence of a variable to identify these cases. However, the author believes that the proportion of self-reports in the survey was minimal in the late 1980s. Forth, this study did not adjust P-values for multiple testing on differences in means of height, weight, and BMI for each group classified by age, sex, and measured BMI. However, the meaningfulness of small differences estimated from the large sample would be more relevant to this study, and the author believes that Cohen’s *d* statistics employed in this study has adequately addressed this issue.

In conclusion, population estimates on nutritional status based on self-reported height and weight were biased among adults in Japan in the late 1980s. Given the lack of a downward trend in the prevalence of adult overweight and obesity in Japan from 1980 to 2013,[26] the importance of accurate measurements of height and weight has been increasing for planning effective programs to control risks for non-communicable diseases. Therefore, it would be sensible to recommend that, in future research, the presence of reporting bias be reassessed through the collection of both self-reported and measured anthropometric data in the national survey.
